# Author Correction: Improving superconductivity in BaFe_2_As_2_-based crystals by cobalt clustering and electronic uniformity

**DOI:** 10.1038/s41598-018-30017-4

**Published:** 2018-07-27

**Authors:** L. Li, Q. Zheng, Q. Zou, S. Rajput, A. O. Ijaduola, Z. Wu, X. P. Wang, H. B. Cao, S. Somnath, S. Jesse, M. Chi, Z. Gai, D. Parker, A. S. Sefat

**Affiliations:** 1 Materials Science and Technology Division, Oak Ridge National Laboratory, Oak Ridge, TN 37831 USA; 2 Center for Nanophase Materials Sciences, Oak Ridge National Laboratory, Oak Ridge, TN 37831 USA; 3 Department of Physics, University of North Georgia, Dahlonega, GA 30597 USA; 4 Fujian Provincial Key Laboratory of Semiconductors and Applications, Collaborative Innovation Center for Optoelectronic Semiconductors and Efficient Devices, Department of Physics, Xiamen University, Xiamen, 361005 P.R. China; 5 Chemical and Engineering Materials Division, Oak Ridge National Laboratory, Oak Ridge, TN 37831 USA; 6 Quantum Condensed Matter Division, Oak Ridge National Laboratory, Oak Ridge, TN 37831 USA; 7 Institute for Functional Imaging of Materials, Oak Ridge National Laboratory, Oak Ridge, TN 37831 USA

Correction to: *Scientific Reports* 10.1038/s41598-017-00984-1, published online 19 April 2017

In Figure 6B, the vertical J_c_ scale is incorrect. The correct Figure 6 appears below as Figure 1.

**Figure 1:**
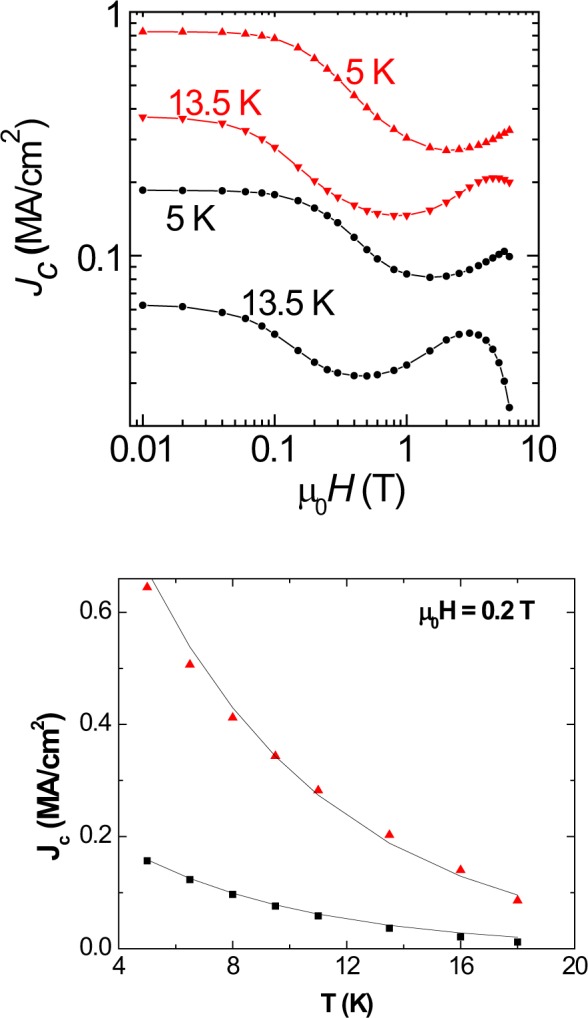
The increase in superconducting critical current density (*J*_*c*_) for annealed optimally-doped Ba(Fe_1−x_Co_x_)_2_ As_2_ crystal. For x = 0.063 and as-grown (in black) and annealed (in red) crystals, (**a**) field dependence of critical current density (*J*_*c*_) below *T*_*c*_, and (**b**) temperature dependence of *J*_*c*_ at 0.2 Tesla.

